# Repetitive transcranial magnetic stimulation for post-stroke epilepsy: a mini-review

**DOI:** 10.1186/s42494-024-00184-1

**Published:** 2024-12-04

**Authors:** Nicholas Aderinto, Gbolahan Olatunji, Emmanuel Kokori, Ikponmwosa Jude Ogieuhi, Abdulrahmon Moradeyo, Owolabi Samuel, Adetola Emmanuel Babalola, Tejiri Napoleon, Wuraola Awosan, Oluwaseun Oyewo, Chidinma Udojike, Oluwatobi Omoworare, Yewande Abigail Adebayo

**Affiliations:** 1 Department of Medicine and Surgery, Ladoke Akintola University of Technology, Ogbomoso, 210214 Nigeria; 2 Department of Medicine and Surgery, University of Ilorin, Ilorin, 240101 Nigeria; 3 Siberian State Medical University, Tomsk, 634050 Russia; 4 Lagos State Health Service Commission, Lagos, 100212 Nigeria; 5 College of Medicine, University of Ibadan, Ibadan, 110115 Nigeria; 6 Liberty University, Lynchburg, VA 24515 USA; 7 Medway Maritime Hospital, Windmill Rd, Gillingham, Kent ME7 5NY, E12000008 UK; 8 College of Medicine, University of Lagos, Lagos, 100212 Nigeria; 9 Department of Medicine and Surgery, Lagos State University, Lagos, 100212 Nigeria; 10 Glangwili General Hospital, Carmarthen, Wales SA31 2AF UK

**Keywords:** rTMS, Repetitive transcranial magnetic stimulation, Post-stroke epilepsy

## Abstract

Post-stroke epilepsy (PSE) is a common complication of stroke, significantly impacting patient’s quality of life. Repetitive transcranial magnetic stimulation (rTMS) is an emerging potential non-invasive treatment for PSE. This review explores current evidence for rTMS in PSE, highlighting its potential benefits and limitations. Initial studies suggested that rTMS may reduce the seizure burden. Some studies observed a trend towards fewer seizures within two weeks of treatment initiation, indicating a relatively rapid response. Additionally, rTMS may be more effective when used in combination with medication, particularly for patients with specific lesion locations (frontal/temporal lobes) and seizure types (complex partial seizures). This points towards the potential of personalized treatment protocols. However, current evidence has limitations. Studies often involve small sample sizes and methodological variations, necessitating larger, well-designed trials with standardized protocols to confirm the efficacy and safety of rTMS in PSE. Future research should also focus on the optimization of treatment parameters, including stimulation frequency, duration, coil placement, and treatment course. Long-term studies are needed to evaluate the persistence of treatment effects on seizure control, cognitive function, and overall patient outcomes. Refining patient selection criteria and investigating the underlying mechanisms of therapeutic effects of rTMS in PSE are also crucial areas for future exploration.

## Background

Stroke is a leading cause of disability and death globally, and is a major risk factor for epilepsy, especially in older adults [1]. It has been estimated that around 0.5% of the global population has epilepsy, with stroke being the most common cause (30–50% of cases) [1–3]. Notably, seizures can occur years after a stroke, or even during its initial stages.

Post-stroke epilepsy (PSE) is characterized by seizures that develop after a stroke [4], being early-onset (occurring within the first two weeks) or late-onset (occurring later) [5]. Early-onset seizures are likely triggered by the initial brain injury, while late-onset seizures are thought to arise from permanent changes in the damaged brain tissue [4–6]. The prevalence of PSE is estimated to be 0.5–2.0%, but reported incidence rates can vary due to the differences in how seizures are diagnosed [7]. While anti-seizure medications are the mainstay in PSE treatment, some patients continue to experience seizures despite medication [8]. This highlights the need for exploring alternative or complementary therapies.

Repetitive transcranial magnetic stimulation (rTMS) is a non-invasive brain stimulation technique that is emerging as a promising tool in treating various neurological conditions including epilepsy [9]. rTMS offers a unique advantage over traditional methods by allowing for targeted stimulation and modulation of neuronal activity. In epilepsy research, rTMS has shown promise as a diagnostic tool to assess medication effectiveness by measuring cortical excitability [10, 11]. The core principle of rTMS involves placing a magnetic coil on the scalp to generate a magnetic field that induces weak electric currents in the targeted brain area, thereby altering neuronal activity patterns [11]. This review explores the potential of rTMS as a treatment for PSE.

## Methodology

We searched PubMed, Cochrane Library, PSYCHInfo, Scopus, and Google Scholar for studies investigating the efficacy and safety of rTMS for treating PSE, published from 2000 to May, 2024. The search strategy incorporated a combination of Medical Subject Headings (MeSH) terms and keywords related to rTMS, PSE, epilepsy, seizure frequency, and seizure severity. Boolean operators (AND, OR, NOT) were used to refine the search and ensure retrieval of relevant studies.

The study inclusion criteria included: (1) recruiting adults diagnosed with PSE, (2) using rTMS for intervention, (3) evaluating the efficacy of rTMS in reducing seizure frequency and/or severity as outcomes, or assessing the safety and tolerability of rTMS for PSE, (4) having a study design of randomized controlled trials (RCTs), observational studies (cohort studies, case-control studies), or case series, and (5) published in English language.

The exclusion criteria included: (1) studies conducted in animal models, (2) techniques other than rTMS were used for epilepsy treatment, and (3) non-peer-reviewed publications (e.g., abstracts, editorials).

Two reviewers (N. A & I. J. O) independently screened the search results based on titles and abstracts. Full-text articles of potentially relevant studies were retrieved and further assessed for eligibility. Any discrepancies between reviewers were resolved through discussion or by consulting a third reviewer (E. K). A narrative synthesis was conducted, summarizing the key findings of the included studies regarding the efficacy and safety of rTMS for PSE.

## Pathophysiology of PSE

Stroke is a major risk factor for epilepsy, particularly in older adults. Seizures after a stroke can occur either during the initial acute phase (early-onset) or years later (late-onset) [8]. The underlying mechanisms of PSE are complex and involve several interrelated processes. Both hemorrhagic and ischemic strokes can cause damage to brain tissue. This damage can include death of neurons and formation of scar tissue. The damaged tissue becomes a source of abnormal electrical activity in the brain, which can lead to seizures [12].

Early-onset seizures that occur within the first two weeks after a stroke, are associated with specific changes in brain chemistry [13]. These changes include excitotoxic release of glutamate that leads to overactivation of neurons. Reduced blood flow and imbalances in electrolytes like calcium and sodium can also contribute to these early seizures [13]. These transient disruptions in brain chemistry can trigger seizures even after the acute stroke phase resolves. Late-onset PSE that develops weeks, months, or even years after a stroke, is a more chronic condition. Permanent structural and functional damage from the stroke progressively worsens, leading to long-term alterations in neuronal activity. These changes result in a hyperexcitable state in the brain, making it more prone to seizures [14–16].

The diagnosis of PSE is made upon the occurrence of two unprovoked seizures (not caused by metabolic, toxic, or other factors) that occur outside the acute stroke phase [17–20]. It is important to note that the diagnosis is not specific for early-onset seizures; late-onset seizures are sufficient for a PSE diagnosis [21–23].

The development of PSE is associated with disturbed balance between excitatory and inhibitory signals within the brain [24]. Following a stroke, the brain generally experiences an increase in excitatory neurotransmission, particularly involving the neurotransmitter glutamate [17, 25]. The increased glutamatergic activity leads to neuronal hyperexcitablility and increased susceptibility to seizures. Furthermore, stroke can disrupt the inhibitory mechanisms in the brain [26], which could be due to the loss of GABAergic neurons, or a decline in the function of GABA receptors [18]. With the lack of adequate inhibition to counterbalance excitation, brain activity becomes dysregulated, significantly increasing the risk of seizures.

## Evidence for the efficacy and safety of rTMS in PSE

A few studies have explored the potential of rTMS as a treatment for PSE (Table 1), suggesting that rTMS may be effective in reducing the seizure frequency, particularly for patients with lesions in specific brain regions. rTMS can decrease the seizure frequency in patients with epilepsy. Brasil-Neto et al. investigated low-frequency rTMS in patients with intractable epilepsy and found a 22.8% reduction in the average daily number of seizures [27]. Other studies with varying stimulation parameters have reported similar reductions in seizure frequency [28–30]. A study by Kinoshita et al. observed a trend towards reduced seizures within two weeks of low-frequency rTMS in patients with medically intractable epilepsy [28], suggesting a potential of early benefit from rTMS treatment in PSE.

**Table 1:** Characteristics of included studies

Study author (year)	Study design	Sample size (*n*)	Stroke type	TMS coil	Frequency (Hz)	Stimulations/Session	Total sessions	Target region	Seizure reduction (%)	Responder rate (%)	Adverse events
Hu et al. (2024) [26]	Randomized controlled trials	121	Ischemic	8-word coil	0.5	1	10	Epileptiform discharges	20	63.49	Headache, scalp discomfort
Tergau et al. (1999) [30]	Open pilot	9	N/A	9-cm round coil	0.33	2	5	Vertex	36.6	77.80	None
Kinoshita et al. (2005) [28]	Clinical trial	7	N/A	9-cm round coil	0.9	2	10	Extra temporal lobe	20.8	57.10	None
Brasil-Neto et al. (2004) [27]	Clinical trial	5	N/A	Round coil	0.3	100	3	Vertex	22.80	11.11	None
Theodore et al. (2002) [29]	Controlled trial	24	N/A	Figure-of-eight	1	2					

Furthermore, a study suggests that rTMS may be more effective in PSE patients with lesions in the frontal, temporal, and parietal lobes [26]. This finding highlights the potential importance of targeting specific brain regions based on the location of the stroke damage. Also, combining rTMS with medication significantly reduces epileptiform discharges and clinical seizures compared to medication treatment alone [26]. This effect is particularly pronounced in patients with temporal and frontal lobe lesions. For patients with parietal lobe lesions, rTMS combined with medication significantly reduces epileptiform discharges. This combinational therapy can also effectively ameliorate depressive symptoms and cognitive impairment, especially in those with frontal lobe lesions.

While rTMS is generally considered safe, some potential adverse effects can occur, particularly during the initial stage of treatment. The most frequent side effect is scalp discomfort or pain at the stimulation site [26–29], manifesting as tingling, itching, or a general feeling of discomfort. In addition, headaches can occur after rTMS sessions, but they are usually mild and can be managed with over-the-counter pain medication [27, 30].

## Discussion

In this paper, we review current evidence for rTMS as a treatment for PSE. While research in this area is ongoing, rTMS has shown potential effectiveness in reducing seizure burden. Some studies observed a potential for early improvement, with a trend towards reduced seizures within just two weeks after treatment initiation [27–29]. This is particularly promising for patients who need a faster response to manage their seizures. Another interesting finding is that the location of stroke damage and the type of seizures may influence the efficacy of rTMS [26, 27]. For instance, rTMS might be more effective for patients with damage in the frontal or temporal lobe, and for those experiencing complex partial seizures. This highlights the importance of developing targeted rTMS protocols that can be tailored for each patient.

The use of low-frequency rTMS for PSE was primarily based on its hypothesized inhibitory effects on the overactive neural circuits implicated in the pathophysiology of the disorder [26–30]. Several preclinical and clinical studies have suggested that low-frequency rTMS over the prefrontal cortex can downregulate cortical excitability, which aligns with the proposed mechanisms underlying PSE [31, 32]. Future studies comparing low-frequency rTMS with other stimulation protocols, such as high-frequency rTMS or theta burst stimulation, are warranted to optimize the treatment outcomes.

Moreover, studies suggest that combining rTMS with medication may be more effective than medication alone in reducing seizure frequency, especially for patients with lesions in the temporal or the frontal lobe [26]. This means that rTMS could be a valuable addition to existing treatment plans, potentially offering extra benefits for patients with medication-resistant PSE. In addition, unlike surgery, which is a more invasive option, rTMS offers a non-invasive approach for PSE patients. Additionally, studies suggest that it is generally well-tolerated, with most side effects being mild and temporary [33]. This makes rTMS a potentially attractive option for patients who experience medication-resistant seizures or who are not suitable candidates for surgery due to underlying health conditions.

While the initial findings on rTMS for PSE are promising, there are limitations that need to be addressed before it can be widely adopted in clinical practice. A consistent theme across studies is the need for larger, well-designed trials with standardized protocols. Current studies often involve relatively small patient groups and methodological variations, making it difficult to definitively confirm the effectiveness of rTMS for PSE. Future studies with larger sample sizes and consistent protocols will be crucial to solidify the evidence base. Also, current research suggests that low-frequency stimulation is generally preferred for PSE. However, this is just a starting point. Further investigations are needed to determine the optimal parameters to maximize the rTMS effectiveness. Additionally, a deeper understanding of the mechanisms by which rTMS works in PSE can pave the way for the development of more targeted and effective treatment approaches.

## Conclusions

Research on rTMS for PSE has provided exciting findings. Initial studies suggest that rTMS helps reduce seizures, showing potential as a valuable tool for managing the condition. The potential benefits are two-fold. First, some studies observed a trend towards fewer seizures within only two weeks of treatment initiation, suggesting a relatively rapid response, which is beneficial for patients needing faster relief. Second, rTMS may have improved efficacy for seizure control when used in combination with medications, particularly in drug-refractory patients. This could greatly improve their quality of life. Another interesting finding is that rTMS might be more effective when targeting certain locations of the stroke damage and types of seizures a patient experiences. This paves the way for personalized treatment.

However, there is still more to learn. Larger studies with standardized protocols are needed to definitively confirm the effectiveness and safety of rTMS. It is also crucial to optimize treatment parameters, such as stimulation frequency and duration. Long-term studies will be essential to assess the lasting impact of rTMS on seizures, cognitive function, and overall patient well-being. Additionally, it is vital to develop more precise criteria for selecting patients who will benefit most from rTMS. Finally, a deeper understanding of how rTMS works in PSE will allow researchers to develop even more targeted and effective treatment approaches. While further investigation is necessary, the initial findings on rTMS for PSE are encouraging. This non-invasive and generally well-tolerated treatment approach holds promise for improving seizure control and quality of life of patients with this condition.

## Data Availability

Data sharing is not applicable to this article as no datasets were generated or analysed during the current study.

## Abbreviations

PSE: Poststroke epilepsy
rTMS: Repetitive transcranial magnetic stimulation

## References

[1] Zhao Y, Li X, Zhang K, Tong T, Cui R. The progress of epilepsy after stroke. Curr Neuropharmacol. 2017;16(1):71–8. 10.2174/1570159X15666170613083253 PMC5771387 28606039

[2] Leonardi M, Ustun TB. The global burden of epilepsy. Epilepsia. 2002;43(s6):21–5. 12190974 10.1046/j.1528-1157.43.s.6.11.x

[3] Olsen TS. Post-stroke epilepsy. Curr Atheroscler Rep. 2001;3(4):340–4. 11389801 10.1007/s11883-001-0029-4

[4] Bladin CF, Alexandrov AV, Bellavance A, Bornstein N, Chambers B, Coté R, et al. Seizures after stroke: a prospective multicenter study. Arch Neurol. 2000;57(11):1617–22. 11074794 10.1001/archneur.57.11.1617

[5] Graham NS, Crichton S, Koutroumanidis M, Wolfe CD, Rudd AG. Incidence and associations of post stroke epilepsy. Stroke. 2013;44(3):605–11. 23370202 10.1161/STROKEAHA.111.000220

[6] Beghi E, Carpio A, Forsgren L, Hesdorffer DC, Malmgren K, Sander JW, et al. Recommendation for a definition of acute symptomatic seizure. Epilepsia. 2010;51(4):671–5. 19732133 10.1111/j.1528-1167.2009.02285.x

[7] Aderinto N, Olatunji G, Muili A, Kokori, E, Edun M, Akinmouju O, et al. A narrative review of non-invasive brain stimulation techniques in neuropsychiatric disorders: current applications and future directions. Egypt J Neurol Psychiatry Neurosurg. 2024;60(50).

[8] Olsen SJ, Patrick M, Hunter SB, Reddy V, Kornstein L, MacKenzie WR, et al. Multistate outbreak of *Listeria monocytogenes* infection linked to delicatessen Turkey meat. Clin Infect Dis. 2005;40(7):962–7. 15824987 10.1086/428575

[9] Tsuboyama M, Kaye HL, Rotenberg A. Review of transcranial magnetic stimulation in epilepsy. Clin Ther. 2020;42(7):1155–68. 32624320 10.1016/j.clinthera.2020.05.016

[10] Valero-Cabré A, Amengual JL, Stengel C, Pascual-Leone A, Coubard OA. Transcranial magnetic stimulation in basic and clinical neuroscience: a comprehensive review of fundamental principles and novel insights. Neurosci Biobehav Rev. 2017;83:381–404. 29032089 10.1016/j.neubiorev.2017.10.006

[11] Klomjai W, Katz R, Lackmy-Vallée A. Basic principles of transcranial magnetic stimulation (TMS) and repetitive TMS (rTMS). Ann Phys Rehabil Med. 2015;58(4):208–13. 26319963 10.1016/j.rehab.2015.05.005

[12] Kuriakose D, Xiao Z. Pathophysiology and treatment of stroke: present status and future perspectives. Int J Mol Sci. 2020;21(20):7609. 33076218 10.3390/ijms21207609 PMC7589849

[13] Myint PK, Staufenberg EFA, Sabanathan K. Post-stroke seizure and post-stroke epilepsy. Postgrad Med J. 2006;82(971):568–72. 16954451 10.1136/pgmj.2005.041426 PMC2585721

[14] Altman K, Shavit-Stein E, Maggio N. Post stroke seizures and epilepsy: from proteases to maladaptive plasticity. Front Cell Neurosci. 2019;13:397. 31607864 10.3389/fncel.2019.00397 PMC6755337

[15] Silverman IE, Restrepo L, Mathews GC. Poststroke seizures. Arch Neurol. 2002;59(2):195–201. 11843689 10.1001/archneur.59.2.195

[16] Sarecka-Hujar B, Kopyta I. Poststroke epilepsy: current perspectives on diagnosis and treatment. Neuropsychiatr Dis Treat. 2018;15:95–103. 30636875 10.2147/NDT.S169579 PMC6309019

[17] Huang L, Xiao W, Wang Y, Li J, Gong J, Tu E, et al. Metabotropic glutamate receptors (mGluRs) in epileptogenesis: an update on abnormal mGluRs signaling and its therapeutic implications. Neural Regen Res. 2024;19(2):360–8. 37488891 10.4103/1673-5374.379018 PMC10503602

[18] van van Hugte EJH, Schubert D, Nadif Kasri N. Excitatory/inhibitory balance in epilepsies and neurodevelopmental disorders: Depolarizing γ-aminobutyric acid as a common mechanism. Epilepsia. 2023;64(8):1975–90. 37195166 10.1111/epi.17651

[19] Fatemi-Ardekani A. Transcranial magnetic stimulation: physics, electrophysiology, and applications. Crit Rev Biomed Eng. 2008;36(5–6):375–412. 20092430 10.1615/critrevbiomedeng.v36.i5-6.30

[20] O’Shea J, Walsh V. Transcranial magnetic stimulation. Curr Biol. 2007;17(6):R196–9. 17371754 10.1016/j.cub.2007.01.030

[21] Ruiz ML, Sarasa MR, Rodríguez LS, Benito-León J, Ristol EG, Arce SA. Current evidence on transcranial magnetic stimulation and its potential usefulness in post-stroke neurorehabilitation: opening new doors to the treatment of cerebrovascular disease. Neurología (English Edition). 2018;33(7):459–72. 10.1016/j.nrl.2016.03.008 27161423

[22] Lefaucheur JP. Transcranial magnetic stimulation. Handb Clin Neurol. 2019;160:559–80. 10.1016/B978-0-444-64032-1.00037-0 31277876

[23] Seshadri S, Wolf PA. Lifetime risk of stroke and dementia: current concepts, and estimates from the Framingham Study. Lancet Neurol. 2007;6(12):1106–14. 18031707 10.1016/S1474-4422(07)70291-0

[24] Starosta M, Cichoń N, Saluk-Bijak J, Miller E. Benefits from repetitive transcranial magnetic stimulation in post-stroke rehabilitation. J Clin Med. 2022;11(8):2149. 35456245 10.3390/jcm11082149 PMC9030945

[25] Corti M, Patten C, Triggs W. Repetitive Transcranial Magnetic Stimulation of Motor Cortex after Stroke. Am J Phys Med Rehabil. 2012;91(3):254–70. 22042336 10.1097/PHM.0b013e318228bf0c

[26] Hu M, Qin B, Li T, Wei C, Su D, Tan Z. Efficacy of rTMS for poststroke epilepsy and its effects on patients’ cognitive function and depressive status. BMC Neurol. 2024;24(1):25. 38216859 10.1186/s12883-024-03531-4 PMC10785375

[27] Brasil-Neto JP, De Araújo DP, Teixeira WA, Araújo VP, Boechat-Barros R. Experimental therapy of epilepsy with transcranial magnetic stimulation: lack of additional benefit with prolonged treatment. Arq Neuropsiquiatr. 2004;62(1):21–5. 15122428 10.1590/s0004-282x2004000100004

[28] Kinoshita M, Ikeda A, Begum T, Yamamoto J, Hitomi T, Shibasaki H. Low-frequency repetitive transcranial magnetic stimulation for seizure suppression in patients with extratemporal lobe epilepsy-a pilot study. Seizure. 2005;14(6):387–92. 16046153 10.1016/j.seizure.2005.05.002

[29] Theodore WH, Hunter K, Chen R, Vega-Bermudez F, Boroojerdi B, Reeves-Tyer P, et al. Transcranial magnetic stimulation for the treatment of seizures: a controlled study. Neurology. 2002;59(4):560–2. 12196649 10.1212/wnl.59.4.560

[30] Tergau F, Naumann U, Paulus W, Steinhoff BJ. Low-frequency repetitive transcranial magnetic stimulation improves intractable epilepsy. Lancet. 1999;353(9171):2209. 10392988 10.1016/S0140-6736(99)01301-X

[31] Han X, Zhu Z, Luan J, Lv P, Xin X, Zhang X, et al. Effects of repetitive transcranial magnetic stimulation and their underlying neural mechanisms evaluated with magnetic resonance imaging-based brain connectivity network analyses. Eur J Radiol Open. 2023;10:100495. 10.1016/j.ejro.2023.100495 PMC10311181 37396489

[32] Chen Y, Cha YH, Li C, Shou G, Gleghorn D, Ding L, et al. Multimodal imaging of repetitive transcranial magnetic stimulation effect on brain network: a combined electroencephalogram and functional magnetic resonance imaging study. Brain Connect. 2019;9(4):311–21. 30803271 10.1089/brain.2018.0647 PMC6533792

[33] Chail A, Saini RK, Bhat PS, Srivastava K, Chauhan V. Transcranial magnetic stimulation: a review of its evolution and current applications. Ind Psychiatry J. 2018;27(2):172–80. 31359968 10.4103/ipj.ipj_88_18 PMC6592198

